# Antibacterial Properties of Flavonoids from Kino of the Eucalypt Tree, *Corymbia torelliana*

**DOI:** 10.3390/plants6030039

**Published:** 2017-09-14

**Authors:** Motahareh Nobakht, Stephen J. Trueman, Helen M. Wallace, Peter R. Brooks, Klrissa J. Streeter, Mohammad Katouli

**Affiliations:** Centre for Genetics, Ecology and Physiology, University of the Sunshine Coast, Maroochydore DC, QLD 4558, Australia; mnobakht@research.usc.edu.au (M.N.); hwallace@usc.edu.au (H.M.W.); pbrooks@usc.edu.au (P.R.B.); kstreete@usc.edu.au (K.J.S.); mkatouli@usc.edu.au (M.K.)

**Keywords:** antibiotic resistance, antimicrobial activity, cytotoxicity, ethnobotany, *Eucalyptus*, natural products, *Pseudomonas aeruginosa*, stingless bees, *Tetragonula*, traditional medicine

## Abstract

Traditional medicine and ecological cues can both help to reveal bioactive natural compounds. Indigenous Australians have long used kino from trunks of the eucalypt tree, *Corymbia citriodora*, in traditional medicine. A closely related eucalypt, *C. torelliana*, produces a fruit resin with antimicrobial properties that is highly attractive to stingless bees. We tested the antimicrobial activity of extracts from kino of *C. citriodora*, *C. torelliana* × *C. citriodora*, and *C. torelliana* against three Gram-negative and two Gram-positive bacteria and the unicellular fungus, *Candida albicans*. All extracts were active against all microbes, with the highest activity observed against *P. aeruginosa*. We tested the activity of seven flavonoids from the kino of *C. torelliana* against *P. aeruginosa* and *S. aureus*. All flavonoids were active against *P. aeruginosa*, and one compound, (+)-(2S)-4′,5,7-trihydroxy-6-methylflavanone, was active against *S. aureus*. Another compound, 4′,5,7-trihydroxy-6,8-dimethylflavanone, greatly increased biofilm formation by both *P. aeruginosa* and *S. aureus*. The presence or absence of methyl groups at positions 6 and 8 in the flavonoid A ring determined their anti-*Staphylococcus* and biofilm-stimulating activity. One of the most abundant and active compounds, 3,4′,5,7-tetrahydroxyflavanone, was tested further against *P. aeruginosa* and was found to be bacteriostatic at its minimum inhibitory concentration of 200 µg/mL. This flavanonol reduced adhesion of *P. aeruginosa* cells while inducing no cytotoxic effects in Vero cells. This study demonstrated the antimicrobial properties of flavonoids in eucalypt kino and highlighted that traditional medicinal knowledge and ecological cues can reveal valuable natural compounds.

## 1. Introduction

The long-term use of antibiotics has led to widespread bacterial resistance, and so there has been a global drive to develop new antibiotics [1,2,3,4]. Traditional medicine based on natural products has led to the discovery of new drugs, and ecological cues can also reveal bioactive natural compounds [5,6,7,8]. Eucalypt kino is a trunk exudate produced by eucalypt trees (*Angophora*, *Corymbia* and *Eucalyptus* spp.) that contains high levels of potentially useful polyphenol compounds. Kino is characterised by its deep rich coloration, high tannin content, polyphenol composition and astringency [9,10]. Indigenous Australians have used kino from *Corymbia* and *Eucalyptus* trees to cure ailments such as diarrhoea, scabies, and haemorrhage [11]. Kino exudates from *Corymbia dichromophloia* trees have also been used as a treatment for toothache, cold and flu, and heart, lung, and bronchial diseases [12]. Kino from *C. citriodora* trees has been used traditionally as a treatment for chronic bowel inflammation [11], and kino from *C. intermedia* trees has been used to treat wounds [13]. Leaf or bark extracts from naturalized *C. torelliana* trees in Nigeria have been used to treat gastrointestinal disorders, wounds, and coughs [14,15].

The biological activity of eucalypt kinos used in traditional medicine has, until recent times, received little attention. Two compounds from *C. citriodora* and *C. maculata* kino, aromadendrin 7-methyl ether and ellagic acid, have long been known to possess antimicrobial activity against the Gram-positive bacterium, *Staphylococcus aureus* [16]. Aqueous kino extracts from 15 eucalypt species have recently been tested for their antimicrobial activity against *S. aureus*, *Bacillus subtilis, Kocuria rhizophila, Pseudomonas aeruginosa, Escherichia coli*, and *Saccharomyces cerevisiae*. Extracts from *C. maculata*, *C. ficifolia*, and *C. calophylla* kino exhibited strong activity against the Gram-positive bacteria, although no activity was observed from any of the eucalypt species against the Gram-negative bacteria [17]. Aqueous and ethanolic extracts from *C. intermedia* leaves also exhibit antimicrobial activity against *S. aureus* and the unicellular fungus, *Candida albicans* [13]. Volatile components from *C. citriodora* essential oil have strong activity against *Mycobacterium tuberculosis* [18]. Extracts of *C. torelliana* leaves or bark have antibacterial activity against a wide range of species including *M. tuberculosis* and non-tuberculous *Mycobacteria* spp., *S. aureus*, *E. coli*, *P. aeruginosa*, *Klebsiella* sp. and *Helicobacter pylori* [14,15,19,20]. *C. torelliana* also has an unusual mutualistic relationship with stingless bees that disperse its seeds and use its fruit resin to construct their nests [21,22,23,24,25,26]. The fruit resin from *C. torelliana* has recently been found to possess antimicrobial properties [27,28,29]. Bees prefer the fruit resin from *C. torelliana* to fruit resin from other species, and this fruit resin may protect their nest from pathogenic microbes [26,27,28]. However, extracts from the kino of *C. torelliana* and *C. citriodora* have not previously been tested for their antimicrobial activity.

**Figure 1:**
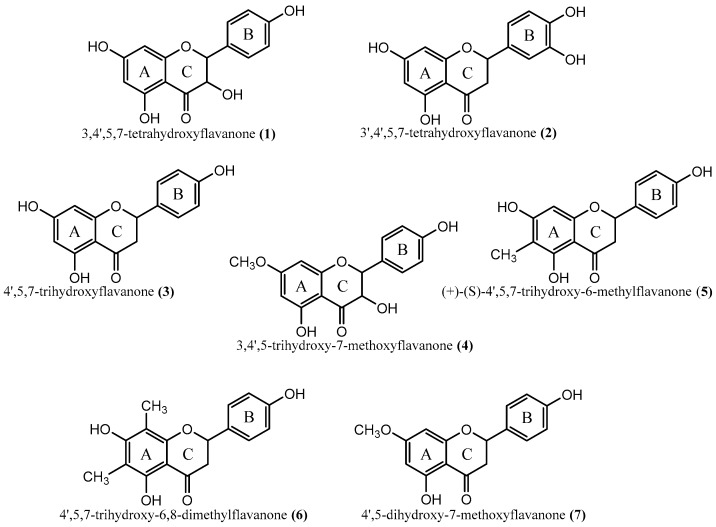
Structures of seven flavonoids isolated from the kino of *Corymbia torelliana*.

In this study, we investigated the antimicrobial activity of extracts from kino of *C. citriodora*, *C. torelliana* and their widely planted hybrid, *C. torelliana* × *C. citriodora*. We examined their activity against three Gram-negative bacteria, *P. aeruginosa*, *E. coli*, and *Salmonella typhimurium*, two Gram-positive bacteria, *S. aureus* and *B. cereus*, and one fungus, *Candida albicans*. Furthermore, we investigated the antibacterial activity against *P. aeruginosa* and *S. aureus* of seven individual flavonoids (Figure 1) isolated from the kino of *C. torelliana* [30]. We also assessed whether the kino extracts and one of the most-abundant and active flavonoids in *C. torelliana* kino had cytotoxic effects.

## 2. Results and Discussion

### 2.1. Antimicrobial Activity and Cytotoxicity of Crude Extracts from Corymbia Trees

**Figure 2:**
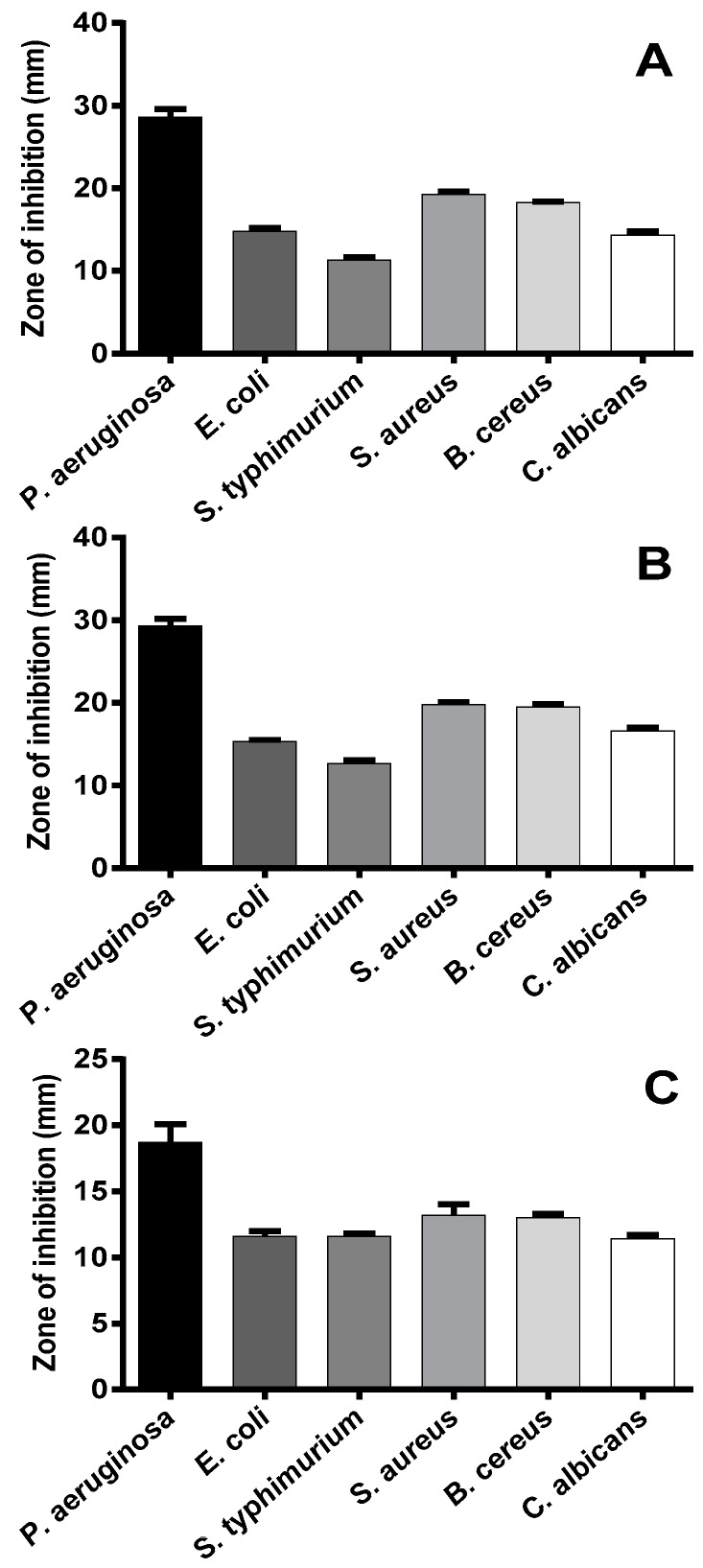
Antimicrobial activity of 400 μg of kino extract from (**A**) *Corymbia citriodora*, (**B**) *C. torelliana* × *C. citriodora*, and (**C**) *C. torelliana* against six microorganisms. Zones of inhibition are presented as mean + S.E. (*n* = 21 trees for *C. citriodora* and the hybrid; *n* = 3 trees for *C. torelliana*). Means among the three kino extracts do not differ significantly (ANOVA, *p* > 0.05).

Ethanolic extracts from kinos of *Corymbia citriodora*, *C. torelliana* × *C. citriodora*, and *C. torelliana* showed strong antimicrobial activity against all of the tested microorganisms (Figure 2). The extent of the inhibition zone varied among the extracts and microorganisms, but the highest inhibition with all three extracts was obtained against *P. aeruginosa*. This is the first report of antimicrobial activity of kino from *C. citriodora*, *C. torelliana* × *C. citriodora* and *C. torelliana* against Gram-negative bacteria, Gram-positive bacteria and *C. albicans*. These results are similar to findings that aqueous kino extracts from the closely related species, *C. maculata*, and the more-distantly related species, *C. ficifolia* and *C. calophylla*, are active against the Gram-positive bacteria, *S. aureus*, *B. subtilis*, and *K. rhizophila* [17]. Aqueous and ethanolic extracts from the leaves of another distantly-related species, *C. intermedia*, also have strong activity against *S. aureus* and *C. albicans* [13]. However, the extracts from *C. maculata, C. ficifolia, C. calophylla*, and *C. intermedia* had little or no activity against *P. aeruginosa* [13,17]*.* The use of aqueous rather than ethanolic extracts [17] and the sampling of leaves rather than kino [13] may explain the lack of activity of other *Corymbia* extracts against Gram-negative bacteria. Alternatively, the differences may be the result of specific chemical compounds that are present in *C. citriodora*, *C. torelliana* × *C. citriodora*, and *C. torelliana* kino.

Different strains of a bacterial species may exhibit different levels of susceptibility to an antimicrobial agent. The highest activity of the crude extracts was against *P. aeruginosa* and so we extended our screening by testing the extracts against four strains of *P. aeruginosa* (i.e., strains C1, C8, C11, and C19) that represented four different clonal groups, isolated recently from clinical cases in our laboratory [31]. The kino extracts showed strong activity against all four strains, except that C11 was resistant to the *C. torelliana* extract (Table 1). This strain was also highly resistant to ticarcillin and intermediately resistant to aztreonam and ticarcillin-clavulanic acid. Ticarcillin is a fourth generation of penicillin, a β-lactam antibiotic. This group of antimicrobial agents inhibits bacteria by penetrating the cytoplasmic membrane and attaching to penicillin binding proteins [32]. Resistance of bacteria to this antibiotic normally develops through a mechanism that inhibits the antibiotic from reaching this target. The resistance of the C11 strain to crude extract of *C. torelliana* suggests that the active component of *C. torelliana* kino might operate by a similar mechanism to these antibiotics. The MIC of kino extracts from *C. citriodora*, *C. torelliana* × *C. citriodora* and *C. torelliana* was 200 µg/mL against each of the bacteria in this study (data not presented). This suggests that, irrespective of the *Corymbia* species, the type and concentration of active compounds in the extracts were similar. At this MIC, 200 µg/mL, the extracts had bacteriostatic activity.

**Figure 3:**
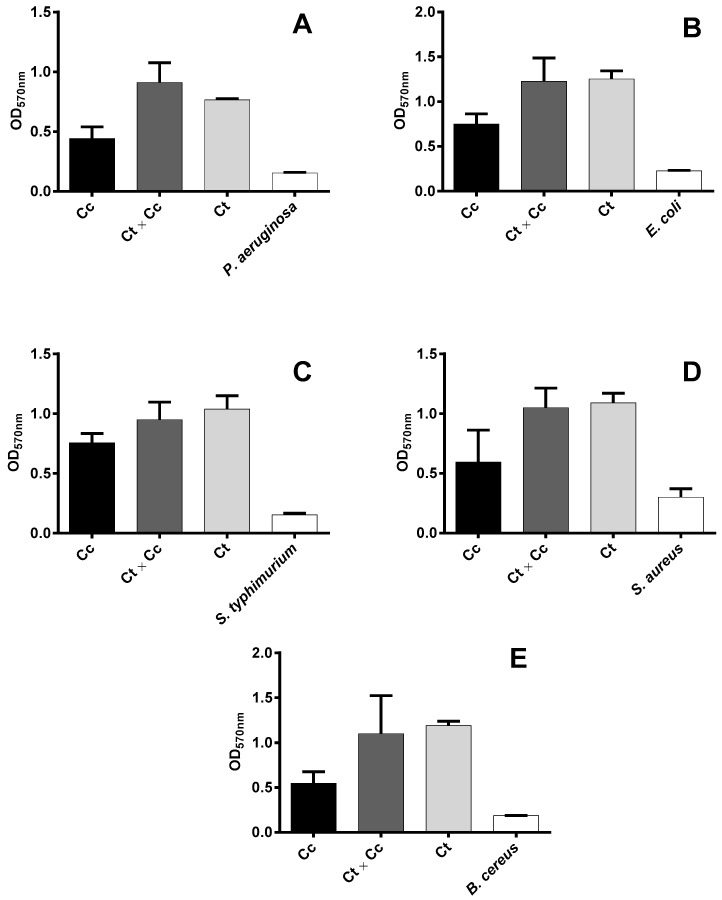
Biofilm formation in the presence of kino extracts from *Corymbia citriodora* (Cc), *C. torelliana* × *C. citriodora* (Ct × Cc), and *C. torelliana* (Ct) by (**A**) *Pseudomonas aeruginosa*, (**B**) *Escherichia coli*, (**C**) *Salmonella typhimurium*, (**D**) *Staphylococcus aureus*, and (**E**) *Bacillus cereus*. Optical densities at 570 nm wavelength (OD_570nm_) are presented as mean + S.E. (*n* = 3 trees for Cc, Ct × Cc, and Ct; *n* = 3 for the kino-free control). Means among the three kino extracts do not differ significantly (ANOVA, *p* > 0.05).

The kino extracts were also tested for their ability to inhibit biofilm formation by the bacteria. All extracts increased biofilm formation of the bacterial strains (Figure 3). These results were unexpected, as we anticipated that extracts capable of inhibiting microbial growth would have also reduced their biofilm formation. However, similar results have been observed during testing of aqueous extracts from the neem tree, *Azadirachta indica*, against two yeast strains [34]. These researchers concluded that increased biofilm formation could be related to an increased level of hydrophobicity, which is a non-specific mechanism for adhesion of bacteria to surfaces. The use of ethanolic extracts could have partly effected the high level of biofilm formation in the current study, as ethanol has potential to increase hydrophobicity. The preparation of aqueous extracts was not feasible due to low solubility of *C. citriodora*, *C. torelliana* × *C. citriodora*, and *C. torelliana* kino in water. From a clinical perspective, biofilm formation is important for survival of bacteria that colonize the host and it assists in physical resistance to phagocytosis and tolerance to antibiotics [35,36]. Under these conditions, antibiotics are prevented from diffusing through the physical barrier formed by the exopolymeric substances in biofilms [37]. Nonetheless, the high antimicrobial activity of all extracts against *P. aeruginosa* indicates the presence of an active compound (or compounds) with anti-*Pseudomonas* activity in the kino from these *Corymbia* species.

The cytotoxicity of *C. citriodora*, *C. torelliana* × *C. citriodora*, and *C. torelliana* kino was tested using 1000 µg of the extracts against Vero cells. No cytopathic effect (CPE), such as detachment or rounding of the cells, was observed. However, cells showed morphological changes, characterized by shrinking, within 48 h. Studies investigating the CPE of bacterial toxins on eukaryotic cells, including Vero cells, have defined cytotoxicity as 50% or more of the cells showing CPE such as rounding or disruption of the cell monolayer within 4 h [38,39,40].

### 2.2. Antibacterial Activity of Flavonoids from Corymbia torelliana

Seven flavonoids isolated from the kino of *C. torelliana* (Figure 1) possessed antibacterial activity against *P. aeruginosa* (Table 2). The activity of these flavonoids against this Gram-negative bacterium is highly significant because the outer membrane of Gram-negative bacteria possesses narrow porin channels that slow the penetration of small hydrophilic solutes and increase their tolerance to antibiotics [41]. Our results suggest that flavonoids from the kino of *C. torelliana* have a mechanism that overcomes this barrier.

Only one of the seven compounds, (+)-(2*S*)-4′,5,7-trihydroxy-6-methylflavanone (**5**), was active against *S. aureus* (Table 2). The other six flavonoids might inhibit *S. aureus* growth at concentrations higher than 50 μg/well, although there can also be relationships between the structure of flavonoids and their antibacterial activity [42]. Hydroxyl groups in the chemical structure increase the activity of flavonoids against methicillin-resistant *S. aureus*, while methoxy groups reduce their activity [43]. However, we did not find the presence of hydroxyl groups to be a defining character in the anti-*Staphylococcus* activity of our flavonoids. For example, (+)-(2*S*)-4′,5,7-trihydroxy-6-methylflavanone (**5**) and 4′,5,7-trihydroxy-6,8-dimethylflavanone (**6**) both have three hydroxyl groups, located at positions 5 and 7 of the A ring and position 4′ of the B ring, while neither compound contains a methoxy group. The unique aspect of compound (**5**) is that it contains a single methyl group, located at position 6 of the A ring. We did not investigate possible relationships between the concentration of flavonoids and their antibacterial effects due to difficulties in obtaining sufficient quantities of flavonoids from *C. torelliana* extracts. However, there can be relationships between the concentration of flavonoids and their antibacterial activity [44].

**Figure 4:**
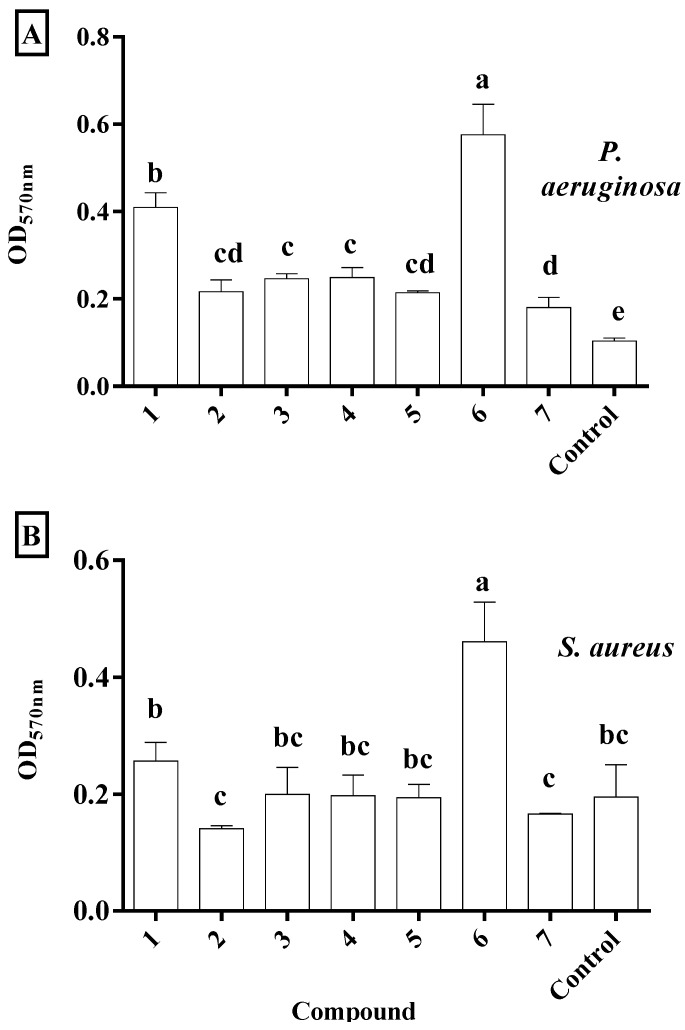
Biofilm formation (OD_570nm_) in the presence of 100 μg of 3,4′,5,7-tetrahydroxyflavanone (1), 3′,4′,5,7-tetrahydroxyflavanone (2), 4′,5,7-trihydroxyflavanone (3), 3,4′,5-trihydroxy-7-methoxyflavanone (4), (+)-(2S)-4′,5,7-trihydroxy-6-methylflavanone (5), 4′,5,7-trihydroxy-6,8-dimethylflavanone (6), and 4′,5-dihydroxy-7-methoxyflavanone (7).

One of the compounds, 4′,5,7-trihydroxy-6,8-dimethylflavanone (**6**), greatly increased biofilm formation by both *P. aeruginosa* and *S. aureus* (Figure 4). This compound is unique in possessing two methyl groups, located at positions 6 and 8 of the A ring (Figure 1). The stimulation of biofilm formation by this flavonoid was highly unexpected. However, extracts of *Azadirachta indica* increase biofilm formation by *C. albicans* [34] and some phenolics and aminoglycosides, at sub-inhibitory concentrations, increase biofilm formation by *P. aeruginosa* and *E. coli* [45,46]. A relationship between the existence of two methyl groups and enhanced biofilm formation has not been reported previously. However, it could be concluded that this phenomenon of increased biofilm formation is entirely, or at least partly, due to a hydrophobicity effect rather than the methyl groups.

### 2.3. Anti-Pseudomonas Activity and Cytotoxicity of 3,4′,5,7-Tetrahydroxyflavanone

**Figure 5:**
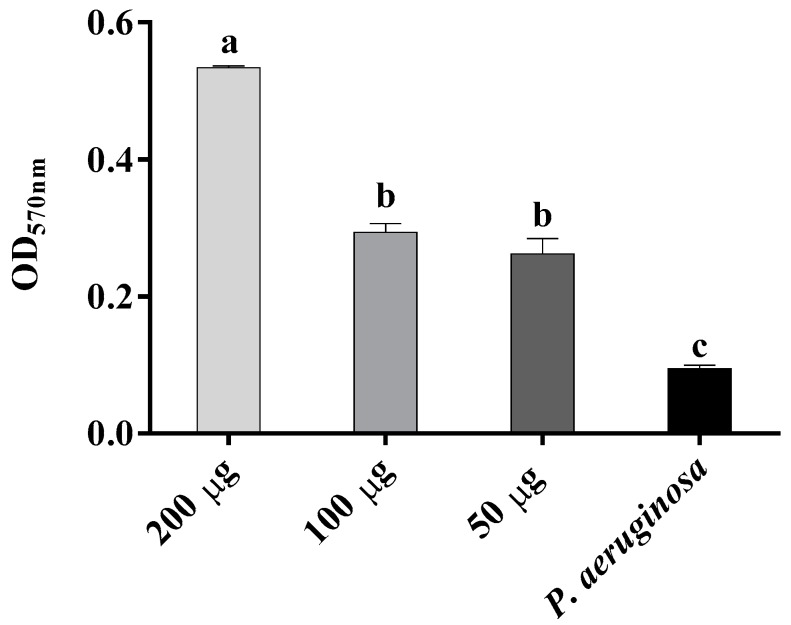
Biofilm formation of *Pseudomonas aeruginosa* in the presence of 200 µg, 100 µg, and 50 µg (final mass) of 3,4′,5,7-tetrahydroxyflavanone from kino exudate of *Corymbia torelliana*. Optical densities at 570 nm wavelength (OD_570nm_) are presented as mean + S.E. Means with different letters are significantly different (ANOVA and Tukey’s HSD test; *p* < 0.05; *n* = 3).

3,4′,5,7-tetrahydroxyflavanone (**1**) inhibited the growth of *P. aeruginosa*, with a minimum inhibitory concentration of 200 µg/mL (Table 3). This compound was bacteriostatic against *P. aeruginosa*. It significantly increased biofilm formation by *P. aeruginosa* at 48 h in comparison with the control, both in this experiment at masses of 200, 100, and 50 µg (Figure 5) and in the previous experiment at a mass of 100 µg (Figure 4). Further investigation is warranted to determine the mechanisms behind the biofilm-stimulating effect of this compound with *P. aeruginosa*. 3,4′,5,7-tetrahydroxyflavanone reduced adhesion of *P. aeruginosa* by 19%, 38%, and 35% at 200 µg, 100 µg, and 50 µg final mass, respectively (Table 3). This is an important result because adhesion to host tissue is an important step in bacterial survival and colonization [47]. The flavonoid induced no cytotoxic effects in Vero cell culture assay after 48 h (Table 3). Crude extracts from *C. torelliana* kino also had no cytotoxic effects on human colorectal epithelial adenocarcinoma (Caco-2) cells (Section 2.1, above). These results, therefore, confirm the potential of 3,4′,5,7-tetrahydroxyflavanone as a new antibacterial agent against *P. aeruginosa*. This is a significant finding because kino extracts have previously been found inactive against Gram-negative bacteria [16,17]. It was concluded that high molecular weight compounds, such as tannins, in the eucalypt kino could not penetrate the outer membrane of Gram-negative bacteria [17]. However, results in the current study suggest that compounds such as 3,4′,5,7-tetrahydroxyflavanone from the kino of *C. torelliana* can overcome this outer membrane barrier. This flavanonol has also been identified in kino from the distantly related species, *C. calophylla* and *C. gummifera* [48,49].

These results confirm that natural products from traditional medicine are promising leads to finding new sources of antibiotic drugs. Natural products have often played a key role in the formulation of new drugs [50] and there is particular interest in polyphenols such as flavonols, flavan-3-ols, and tannins for their potential antimicrobial effects [51]. Indigenous Australians have used kino exudates from eucalypt trees including *C. citriodora* to cure various ailments, and eucalypt kinos are well known for their polyphenol content [11]. The successful discovery of new sources of drugs based on traditional knowledge can also be guided by ecological cues [7]. *C. torelliana* is a geographically-restricted species that is closely related to the traditional medicinal species, *C. citriodora*, but which has a unique mutualism with stingless bees [21]. These bees are strongly attracted to the resin of *C. torelliana* fruits, which they use to construct their nests, and the bees disperse the seeds of this species [22,23,24,25,26]. Resin from the fruit of *C. torelliana* has also been shown to possess antimicrobial properties [27,28]. We were, therefore, guided by both traditional knowledge of the medicinal properties of this eucalypt group and ecological information on the attractiveness to bees of one particular species in this group, to identify potentially valuable antibacterial compounds.

## 3. Materials and Methods

### 3.1. Antimicrobial Activity and Cytotoxicity of Crude Extracts from Corymbia Trees

Fresh kino samples were obtained from *C. citriodora* subsp. *variegata* and *C. torelliana × C. citriodora* subsp. *variegata* (21 trees each) in a forestry plantation at Binjour (25°30′ S, 151°27′ E), Australia, established by the Queensland Department of Agriculture and Fisheries [52,53]. Samples were also collected from three *C. torelliana* trees on the Sunshine Coast (26°42′ S, 153°02′ E), Australia. *C. citriodora*, and hybrids between *C. citriodora* and *C. torelliana*, are grown extensively in forestry plantations [54,55,56,57,58,59,60] but *C. torelliana* is rarely grown in plantations, partly because of its invasive potential [21,25]. Therefore, the three samples of *C. torelliana* were obtained from isolated trees in amenity plantings. Kino samples were collected from naturally-occurring, freely-flowing trunk exudates into clean vials, transported to the laboratory on ice, and stored in the dark at −20 °C until testing. Collection of samples from older, crystallized exudates was avoided due to the effect of long-term sunlight exposure on the chemical composition of kino [61].

Crude extracts (1.0%, w/v) were prepared from the kino of each tree in ethanol (70%, v/v) at laboratory temperature and filtered through cotton wool to remove coarse debris. The kino extracts were then examined for their antimicrobial activity using well-diffusion methods [62,63]. Microbial suspensions were prepared by inoculating single colonies of the type culture strains, *P. aeruginosa* (ATCC 27853), *E. coli* (ATCC 25922), *S. typhimurium* (ATCC 13311), *S. aureus* (ATCC 25923), *B. cereus* (ATCC 11788), and a laboratory strain of *Candida albicans* into tryptone soya broth (TSB) (Oxoid, Australia). Cultures were incubated overnight at 37 °C on a rotary shaker (150 rpm). The bacterial suspensions were diluted using phosphate buffered saline (PBS, pH = 7.4) to approximately 1.0 × 10^9^ colony forming units (cfu)/mL using a spectrophotometer at 600 nm wavelength. From each bacterial suspension, 2.5 mL was transferred to 247.5 mL of molten Mueller-Hinton agar at approximately 55°C, and thoroughly mixed to obtain a uniform concentration of 1.0 × 10^7^ cfu/mL. Each of the six agar suspensions was poured into one sterilized acrylic plate (30 cm × 30 cm internal diameter and 1 cm depth) and allowed to set. Using a sterile cork borer of 4 mm external diameter, 49 holes were cut in each plate and the holes were inoculated with 40 µL of a 1.0% (w/v) solution of kino from each tree, providing a final mass of 400 µg of kino in each well. Plates were covered and incubated for 18 h at 37 °C, and the zone of inhibition for each kino extract was measured. A zone of inhibition >5 mm was considered as positive [64]. Ethanol (70%, v/v) was used as a negative control, and this provided no growth inhibition in any experiment. We also used *P. aeruginosa* (ATCC 27853) as the positive control for testing four clinical strains of *P. aeruginosa* (see below).

Based on the initial antimicrobial activity results, 40 µL of a 1.0% (w/v) solution of kino (final mass of 400 µg) from crude extract of *C. citriodora*, *C. torelliana* × *C. citriodora* or *C. torelliana* was also tested against four wild clinical strains of *P. aeruginosa*, representing different clonal groups. These strains were obtained from a study investigating the clonality of *P. aeruginosa* strains and their virulence properties [31]. The four clinical strains were also tested for their resistance to twelve antimicrobial agents according to Clinical Laboratory Standard Institute (CLSI) guidelines [33], and the results were compared with the antimicrobial activities of the kino extracts. The antimicrobial impregnated disks (Oxoid) included piperacillin (100 µg), ticarcillin (75 µg), piperacillin–tazobactam (100/10 µg), ticarcillin–clavulanic acid (75/10 µg), ceftazidime (30 µg), cefepime (30 µg), aztreonam (30 µg), imipenem (10 µg), gentamicin (10 µg), amikacin (30 µg), ciprofloxacin (5 µg) or norfloxacin (10 µg). Disks were placed on Mueller-Hinton agar that had been inoculated with bacterial suspension at a concentration of 1.0 × 10^7^ cfu/mL. Plates were then incubated at 37 °C for 16–18 h, after which the diameter of the inhibition zone was measured. The strains were classified as susceptible (S), intermediate (I) or resistant (R) to the antibiotics according to CLSI guidelines [33].

MIC values of the extracts were measured using standard methods [65]. Fresh cultures of the microorganisms were prepared and added to Mueller-Hinton broth to give a final concentration of 1.0 × 10^7^ cfu/mL. Five concentrations of kino samples (1.0%, 0.8%, 0.5%, 0.3%, and 0.1%; w/v) were prepared from three trees of each taxon and tested against each microorganism, giving final masses of 400 µg, 320 µg, 200 µg, 120 µg, and 40 µg of kino extract per tube. The tubes were incubated on a rotary shaker (150 rpm) at 37 °C for 18 h, and the growth of the microorganisms was recorded visually. A loopful of the last tube showing no growth was then subcultured on tryptone soy agar (TSA) to determine whether the kino was bactericidal or bacteriostatic. Plates were incubated at 37 °C for 18 h.

Biofilm formation in the presence of kino was tested in 96-well tissue culture plates. Suspensions of bacteria grown on TSA agar were prepared to a final concentration of 1.0 × 10^6^ cfu/mL. As a control, an aliquot of 200 µL of each suspension was added to a well of the plate and incubated without shaking for 48 h at 37 °C. The highest concentration of prepared kino extract (i.e. 1.0%, w/v) from three trees of each taxon was used for biofilm formation assay. 100 µL of sample was added to 100 µL of TSB in each well, and bacterial growth was measured at 600 nm wavelength before staining. The growth medium was removed and plates were rinsed twice with PBS to rinse away non-adhering bacteria. The plates were then allowed to dry. Bacterial biofilm in the wells was stained with 220 μL of crystal violet (0.3%) for 10 min, excess stain was removed with tap water, and the dye was solubilised using 250 μL of acetone/ethanol (20/80, v/v) by shaking at 200 rpm for 10 min. Dissolved crystal violet was measured at 570 nm wavelength. The kino samples from each tree were tested in triplicate. The average optical density (OD) of all three wells at 570 nm wavelength was calculated.

Cytotoxicity of the crude extracts was tested against Vero cells (ATCC CCL-81) derived from African Green Monkey kidneys. Cells were maintained at 37 °C and 5% CO_2_ in Eagle’s minimal essential medium (EMEM) (Lonza, Australia) supplemented with 10% foetal bovine serum (FBS; Lonza) and 1% penicillin/streptomycin solution (Lonza). 200 µL of cell suspension was seeded into 96-well tissue culture plates and grown to 100% confluence in EMEM without antibiotics. Three concentrations of compound (200 µg, 100 µg, and 50 µg) were tested and all tests were performed in triplicate. Cells were visually examined for cytotoxic effects after 4, 24, and 48 h using an inverted phase contrast microscope (×400). The kino extracts were deemed cytotoxic if cell rounding and cell death (detachment from the bottom of wells) occurred in more than 50% of cells [38]. 

### 3.2. Antibacterial Activity of Flavonoids from Corymbia torelliana 

Fresh kino samples from *C. torelliana* trees on the Sunshine Coast (see Section 3.1, above) were extracted in ethyl acetate/water (4/3; v/v). The extracts were stored at -20°C until fractionation by preparative HPLC. The dry extract (100 mg) was dissolved in acetonitrile/water (1/1; v/v) for fractionation by preparative chromatography. Seven flavonoids were recovered and identified by spectroscopic and spectrometric methods including UV, 1D, and 2D NMR, and UPLC-HR-MS, as described previously [30]: 3,4′,5,7-tetrahydroxyflavanone (**1**), 3′,4′,5,7-tetrahydroxyflavanone (**2**), 4′,5,7-trihydroxyflavanone (**3**), 3,4′,5-trihydroxy-7-methoxyflavanone (**4**), (+)-(2S)-4′,5,7-trihydroxy-6-methylflavanone (**5**), 4′,5,7-trihydroxy-6,8-dimethylflavanone (**6**), and 4′,5-dihydroxy-7-methoxyflavanone (**7**) (Figure 1). Two of these compounds, (**1**) and (**4**), are flavanonols, while the other compounds are flavanones.

Each flavonoid was prepared in ethanol (70%; v/v) at a final mass of 50 µg and tested for its antibacterial activity against *P. aeruginosa* (ATCC 27853) and *S. aureus* (ATCC 25923) using a well-diffusion method [63]. Bacterial suspensions were prepared in PBS (1.0 × 10^9^ cfu/mL) after overnight growth in TSB. Molten Mueller-Hinton agar was inoculated with bacterial suspension to give a concentration of 1.0 × 10^7^ cfu/mL, and then allowed to set. Holes cut in the plate were inoculated with 50 µL of a 0.1% (w/v) solution of each flavonoid, providing a final mass of 50 µg in each well. This mass was chosen due to the concentration of pure compounds available after several rounds of extraction. Plates were covered and incubated for 18 h at 37 °C, and the zone of inhibition was measured. Ethanol (70%; v/v) provided no growth inhibition in any experiment. No differences in solubility in ethanol were observed among the seven flavonoids.

Anti-biofilm activity of the seven flavonoids was tested in 96-well tissue culture plates using *P. aeruginosa* (ATCC 27853) and *S. aureus* (ATCC 25923). Fresh bacterial suspensions (1.0 × 10^6^ cfu/mL) were prepared in PBS (pH 7.4), with their concentrations determined by observing the OD_600_ value. This suspension was diluted 1:100 in sterile TSB, and 200 μL of suspension was inoculated into a sterile 96-well plate, avoiding the outermost wells to minimise the possibility of desiccation. 100 µL of each flavonoid solution (100 µg of compound) was added to 100 µL of TSB (diluted 1:50) inoculated with bacteria. The plates were processed and assessed for biofilm formation using the method described in Section 3.1 (above). All tests were performed in triplicate wells. The mean (±standard error) zone of inhibition or optical density of the three wells was calculated for each sample.

### 3.3. Anti-Pseudomonas Activity and Cytotoxicity of 3,4′,5,7-tetrahydroxyflavanone

One of the most-abundant and active flavonoids, 3,4′,5,7-tetrahydroxyflavanone (**1**), was tested further against one of the most susceptible bacteria. *P. aeruginosa* (ATCC 27853) was grown and maintained in TSB. Cultures were incubated overnight at 37 °C on a rotary shaker (150 rpm). MICs were measured using methods described previously [65]. Fresh cultures of each bacterium were prepared and added to Mueller-Hinton broth to give a final concentration of c. 1.0 × 10^7^ cfu/mL. Three concentrations of the flavonoid were prepared: 200 µg, 100 µg, and 50 µg per mL. Bacteria were grown at 100 rpm at 37 °C for 18 h, and the presence or absence of growth was determined visually. The last tube showing no growth was then subcultured on TSA plates to determine whether the MIC was bactericidal or bacteriostatic. These plates were incubated at 37 °C and the growth or lack of growth was observed after 18 h.

Antibiofilm activity of 3,4′,5,7-tetrahydroxyflavanone was tested in 96-well tissue culture plates against the same bacterial strains (above). Fresh bacterial suspensions (1.0 × 10^6^ cfu/mL) were prepared in sterile PBS (pH 7.4). This suspension was diluted 1:100 in sterile TSB and 200 μL was inoculated into a sterile tissue culture plate, avoiding the outermost wells. Each plate included positive controls (bacteria without any compounds) and negative controls (only TSB broth). 100 µL of each flavonoid solution (to reach 200 µg, 100 µg, and 50 µg final mass) was added to 100 µL of TSB (diluted 1:50) inoculated with bacterium. The plates were processed and assessed for biofilm formation using the method described in Section 3.1 (above). All tests were performed in triplicate and the average OD was calculated.

The ability of *P. aeruginosa* to adhere to Caco-2 cells (derived from human colon adenocarcinoma) was tested in the presence and the absence of 3,4′,5,7-tetrahydroxyflavanone. Cells were grown on glass coverslips (12 mm diameter, 1 mm thick) to 75% confluence in EMEM supplemented with 10% FBS and 1% penicillin/streptomycin in a 24-well culture plate (Nunc, Australia) at 37 °C in 5% CO_2_. Cells were rinsed three times with 1 mL of EMEM to remove residual antibiotics, and the medium was replaced with antibiotic-free culture medium. Bacteria were grown in TSB at 110 rpm for 4 h at 37 °C. Bacterial suspensions were centrifuged at 3000 rpm for 10 min and the bacterial pellets were resuspended in sterile PBS (pH 7.4) and adjusted to a concentration of c. 1 × 10^9^ cfu/mL. 100 μL of bacterial suspension was inoculated per well, and the plates were incubated at 37 °C in 5% CO_2_ for 90 min. Non-adherent bacteria were removed by washing the cells three times with sterile PBS. Cells were fixed with 95% ethanol for 5 min, air dried, Gram-stained, and examined by light microscopy (×1000). Bacterial adhesion was assessed [66], with the percentage of adherent bacteria determined by the presence of bacteria on 100 randomly selected cells, and the degree of bacterial adhesion assessed by counting the number of attached bacteria on 25 randomly selected Caco-2 cells. Strains that adhered to <10% of Caco-2 cells were deemed non-adherent. These tests were performed in duplicate. Cytotoxicity of 3,4′,5,7-tetrahydroxyflavanone was tested in vitro against Vero cells as described above. 200 µL of cell suspension was seeded into 96-well tissue culture plates and grown to 100% confluence in medium without antibiotics. The flavonoid was tested at 200 µg, 100 µg and 50 µg final mass, with the tests performed in triplicate. Cells were examined for cytotoxic effects after 4, 24 and 48 h using and the cytotoxicity was interpreted as described above. These tests were performed in triplicate.

### 3.4. Data Analysis

Data were analysed by analysis of variance (ANOVA) followed by post-hoc Tukey’s Honestly Significant Difference (HSD) test when the ANOVA detected significant differences (*p* < 0.05) among the means.

## Figures and Tables

**Table 1:** Antimicrobial activity of a 1.0% (v/v) solution of kino extract from *Corymbia citriodora*, *C. torelliana* × *C. citriodora*, or *C. torelliana* against four clinical strains of *Pseudomonas aeruginosa* (C1, C8, C11, and C19) representing different clonal types*.* Antibiotic susceptibility profile of isolates is also given*.*

*Corymbia* Species or Hybrid	Strain/Zone of Inhibition (mm) *			
	C1	C8	C11	C19
*C. citriodora*	12 ± 0	18 ± 1	15 ± 1	11 ± 0
*C. torelliana × C. citriodora*	11 ± 0	16 ± 1	19 ± 1	12 ± 0
*C. torelliana*	11 ± 0	11 ± 1	1 ± 1 **	12 ± 0
**Antibiotic Susceptibility Profile** †				
Amikacin (30 µg)	S	S	S	S
Aztreonam (30 µg)	S	I	I	I
Ceftazidime (30 µg)	S	S	S	S
Cefepime (30 µg)	S	S	S	S
Piperacillin (100 µg)	S	I	S	R
Piperacillin-tazobactam (100/10 µg)	S	R	S	I
Ticarcillin (75 µg)	I	I	R	I
Gentamicin (10 µg)	S	I	S	S
Ciprofloxacin (5 µg)	S	S	S	S
Norfloxacin (10 µg)	S	S	S	S
Imipenem (10 µg)	R	S	S	S
Ticarcillin-clavulanic acid (75/10 µg)	I	R	I	R

* Zones of inhibition are presented as mean ± S.E. (*n* = 9 trees for *C. citriodora* and the hybrid; *n* = 3 trees for *C. torelliana*). Means within a *P. aeruginosa* strain do not differ significantly (ANOVA, *p* > 0.05); ** Distance (mm) from the rim of the well; † Antibiotic susceptibility profile is classified as susceptible (S), intermediate (I) or resistant (R) according to CLSI guidelines [33].

**Table 2:** Antimicrobial activity of seven flavonoids [3,4′,5,7-tetrahydroxyflavanone (**1**), 3′,4′,5,7-tetrahydroxyflavanone (**2**), 4′,5,7-trihydroxyflavanone (**3**), 3,4′,5-trihydroxy-7-methoxyflavanone (**4**), (+)-(2S)-4′,5,7-trihydroxy-6-methylflavanone (**5**), 4′,5,7-trihydroxy-6,8-dimethylflavanone (**6**), and 4′,5-dihydroxy-7-methoxyflavanone (7)] from *Corymbia torelliana* kino against *Pseudomonas aeruginosa* and *Staphylococcus aureus*.

Bacterium	Zone of Inhibition (mm)						
	Compound						
	1	2	3	4	5	6	7
*P. aeruginosa*	20.3 ± 1.8	6.7 ± 6.7	19.7 ± 1.9	12.3 ± 6.3	18.7 ± 1.2	24.7 ± 2.9	20.3 ± 2.8
*S. aureus*	inactive	inactive	inactive	inactive	12.7 ± 1.8	inactive	inactive

Means (± S.E.) among seven flavonoids within *P. aeruginosa* do not differ significantly (ANOVA, *p* > 0.05).

**Table 3:** Minimum inhibitory concentration (MIC) and reduction in adhesion of *Pseudomonas aeruginosa* in the presence of 3,4′,5,7-tetrahydroxy-flavanone from *Corymbia torelliana* kino.

MIC (µg/mL)	Control Adhesion cfu (Mean ± S.E.)	Adhesion Difference (%)		
		Final Mass (µg)		
		200	100	50
200	3.86 ± 0.16	−19	−38	−35
